# Psychoanalytic and Psychodynamic Approaches to Skin Disease and Their Implications for Dermatology

**DOI:** 10.1111/srt.70176

**Published:** 2025-05-05

**Authors:** Anna Dattolo, Isabella J. Tan, Mohammad Jafferany

**Affiliations:** ^1^ IDI‐Istituto Dermopatico dell'Immacolata Rome Italy; ^2^ Robert Wood Johnson Medical School Rutgers the State University of New Jersey Piscataway New Jersey USA; ^3^ Department of Psychiatry and Behavioral Sciences Central Michigan University College of Medicine Mount Pleasant Michigan USA

**Keywords:** mind‐skin connection, psychoanalysis, psychodermatology, psychodynamic

## 1 Background

The skin serves as a critical interface between the internal and external worlds, reflecting emotional states while acting as a physical barrier. Dermatology must increasingly acknowledge the complex psychodynamic interactions influencing disease manifestation, as chronic skin conditions like psoriasis, atopic dermatitis, and alopecia areata demonstrate significant psychosomatic components [1, 2]. Integrating psychoanalysis and psychodynamic theories into dermatological practice can provide a more comprehensive approach to patient care by addressing both the physical and emotional dimensions of these disorders [1, 2].

### 1.1 The Role of Skin in Emotional Regulation

The skin plays a foundational role in emotional and identity development, a concept emphasized in Didier Anzieu's theory of the “Ego Skin,” which posits that the skin functions as a psychological container for emotions [3]. Early life experiences, such as skin‐to‐skin contact with caregivers, influence the regulation of emotions and self‐perception, with disruptions in these early interactions potentially contributing to later dermatological manifestations. Conditions such as atopic dermatitis, which frequently present in infancy, may reflect underlying emotional dysregulation [3].

Evidence supports the role of early touch deprivation in the dysregulation of stress response mechanisms, leading to increased vulnerability to both psychological and dermatological disorders [3]. Psychological stress is a well‐established trigger for exacerbations of chronic skin diseases, and patients frequently report worsening of symptoms during periods of heightened emotional distress. This stress‐skin axis highlights the necessity of addressing emotional factors in dermatological management [4].

### 1.2 Psychodermatology: Integration of Mind and Skin

Psychodermatology underscores the bidirectional link between psychiatric disorders and skin disease, with stress playing a pivotal role in triggering or exacerbating conditions like psoriasis, vitiligo, and hidradenitis suppurativa (Table 1). Biologically, stress activates the hypothalamic‐pituitary‐adrenal (HPA) axis, releasing cortisol and pro‐inflammatory cytokines that exacerbate skin inflammation [5]. Stress‐induced neuroimmune interactions, involving neuropeptides and immune system modulation, further contribute to dermatologic disease pathogenesis [5]. Psychodynamically, unresolved emotional conflicts may manifest through the skin as a somatic outlet for psychological distress [5].

**TABLE 1:** Psychodermatological conditions and associated psychological factors.

Dermatological condition	Psychological factor(s)	Relevant psychodynamic concept	Key psychodynamic treatment approaches
Psoriasis	Depression, anxiety	Ego‐skin dysfunction	Psychodynamic psychotherapy, stress management
Atopic dermatitis	Emotional dysregulation, stress	Emotional skin as a boundary	Cognitive‐behavioral therapy (CBT), emotion regulation
Alopecia areata	Helplessness, anger, shame	Containment and identity issues	Psychodynamic therapy, self‐esteem building
Hidradenitis suppurativa	Depression, alexithymia	Fragmentation and repression	Psychoanalysis, emotional expression techniques
Vitiligo	Social anxiety, identity confusion	Skin as container of self	Psychodynamic psychotherapy, identity‐focused therapy

By viewing the skin as a “container” for unresolved emotions, dermatologists can gain deeper insight into the psychogenic factors contributing to skin disease. This approach calls for an interdisciplinary framework that incorporates dermatologists, psychiatrists, and psychologists working collaboratively to address the psychological and somatic components of dermatological disorders.

### 1.3 Clinical Implications

Despite established psychodynamic and psychoanalytical associations in dermatological conditions, psychodermatology is underutilized in clinical practice [1]. Integrating psychodynamic and psychoanalytical principles may be instrumental in improving diagnostic accuracy and therapeutic efficacy [5]. Incorporating psychiatric treatments, such as cognitive behavioral therapy, stress management, and emotional regulation techniques, alongside conventional dermatologic care, enables a comprehensive approach to addressing the emotional underpinnings of skin disorders.

Dermatologists should explore patients' psychological histories, as emotional trauma and abuse, sexual abuse in childhood, stress, and other psychosocial factors influence disease onset, exacerbation, and treatment resistance. Collaboration with mental health professionals should be routine in managing complex dermatological cases, particularly those with a substantial psychosomatic burden.

Integrating psychodynamic principles into dermatology addresses the complex interplay between emotional and physiological factors in skin disease. Dermatologists can explore CME courses, psychoanalytic institute workshops, and interdisciplinary collaborations to gain relevant training. This approach enhances holistic care and improves patient outcomes.

## Conflicts of Interest

The authors declare no conflicts of interest.

## Data Availability

The data that support the findings of this study are available from the corresponding author upon reasonable request.
